# Prof. Paul E. Eve, M. D.

**Published:** 1877-12

**Authors:** 


### PROF. PAUL F. EVE, M. D.,

NASHVILLE, TENN.

Professor Paul Fitzsimmons Eve, the distinguished Southern surgeon, died suddenly, while in attendance upon a patient, November 3d, aged seventy-one years. He was born June 26, 1806, near Augusta, Georgia; graduated at the University of Georgia in

1826; as M. D., at the University of Pennsylvania in 1828, and was a student several years in Europe. He served as a volunteer surgeon in the Polish revolution of 1831, and received therefor the Golden Cross of Honor of Poland that year. He became Professor of Surgery in the Medical College of Georgia in 1832, in the Louisville University in 1849, in the Nashville University in 1850, and in the Missouri Medical College, St. Louis, in 1868. In 1870 he accepted the chair of Operative and Clinical Surgery in the University of Nashville, which position he held at the time of his death. As a representative man for the South he was, in 1857, chosen President of the American Medical Association. During the rebellion he served as surgeon in the Confederate Army, and for the greater part of his professional career was identified, directly or indirectly, with medical journalism in his section of country. A hard worker in his profession, his strictly methodical and temperate habits enabled him to carry his burden of duties up to the very threshold of death, and lay them down to enter at once into his eternal rest. Prof. Eve, as a surgeon, will be best remembered in connection with his remarkable successes as a lithotomist. Of ninety-two bilateral operations for stone eight only terminated fatally. His last notable contribution to medical literature was his address on Surgery at the International Medical Congress in 1876.—*Medical Record.*

